# Sulfonyl Imide Acid-Functionalized Membranes via Ni (0) Catalyzed Carbon-Carbon Coupling Polymerization for Fuel Cells

**DOI:** 10.3390/membranes11010049

**Published:** 2021-01-12

**Authors:** Sabuj Chandra Sutradhar, Sujin Yoon, Taewook Ryu, Lei Jin, Wei Zhang, Hohyoun Jang, Whangi Kim

**Affiliations:** 1 Department of Applied Chemistry, Konkuk University, Chungju 27478, Korea; sabujchandra@gmail.com (S.C.S.); ysj920126@naver.com (S.Y.); gundam0924@naver.com (T.R.); jinlei8761@naver.com (L.J.); arno_zw@hotmail.com (W.Z.); 2 Department of Liberal Art, Konkuk University, Chungju 27478, Korea; 200417450@kku.ac.kr

**Keywords:** sulfonyl imide, C-C coupling, nickel catalyst, super gas-phase acidity, morphology

## Abstract

Polymer membranes, having improved conductivity with enhanced thermal and chemical stability, are desirable for proton exchange membranes fuel cell application. Hence, poly(benzophenone)s membranes (SI-PBP) containing super gas-phase acidic sulfonyl imide groups have been prepared from 2,5-dichlorobenzophenone (DCBP) monomer by C-C coupling polymerization using Ni (0) catalyst. The entirely aromatic C-C coupled polymer backbones of the SI-PBP membranes provide exceptional dimensional stability with rational ion exchange capacity (IEC) from 1.85 to 2.30 mS/cm. The as-synthesized SI-PBP membranes provide enhanced proton conductivity (107.07 mS/cm) compared to Nafion 211^®^ (104.5 mS/cm). The notable thermal and chemical stability of the SI-PBP membranes have been assessed by the thermogravimetric analysis (TGA) and Fenton’s test, respectively. The well distinct surface morphology of the SI-PBP membranes has been confirmed by the atomic force microscopy (AFM). These results of SI-PBP membranes comply with all the requirements for fuel cell applications.

## 1. Introduction

Eco-friendly proton exchange membranes fuel cells (PEMFC) are regarded as an effective substitute for fossil fuels [1,2,3,4,5,6]. In PEMFC, the polymer electrolyte membranes (PEM) plays a vital role by converting the H_2_ and oxidant into electrical energy. Therefore, PEM materials are the core research area for fuel cells. Although polyfluorosulfonic acid (PFSA) based membranes, Nafions^®^, are considered as a standard commercial material for proton exchange membranes (PEM), high cost, harsh manufacturing process, and difficulties for structural modification limit its applications [7,8,9]. Aromatic hydrocarbon-based polymers with different polymer backbones [10,11,12] have already been studied as an alternative to PFSA-based membranes. Comparatively, the pre-sulfonation process (i.e., using sulfonated monomers) is found to be more convenient than post-sulfonation of the membranes but sometimes it can be very difficult to achieve the high molecular weight polymers from primarily sulfonated monomers [12,13,14,15,16]. Additionally, most of these sulfonated polymers show oxidative and hydrolytic stabilities either for their ether linkage-based polymer backbones nor for the attachment of sulfonic acid groups directly to the main polymer chain as they are susceptible to nucleophilic attacks [17,18]. Typically, the lower acidity of the sulfonic acid groups is also accountable for the poor conductivity in the fuel cells. Therefore, researchers have attempted to find out the alternative acid functional groups with nonfluorinated or partially fluorinated membranes other than perfluorosulfonic acids (PFSA) [19]. Recently, sulfonyl imide membranes have become the main interest in their super gas-phase acidity and exceptional thermal stability [20,21]. It is claimed that higher charge delocalization capability is responsible for its higher acidity [22,23,24,25,26,27,28,29,30]. As a result, researchers reported some polymer membranes using sulfonyl imide groups [31,32,33,34,35,36,37,38,39] and demonstrated as potential candidates for fuel cell applications [40,41,42,43]. Most of the sulfonyl imide membranes also show poor chemical stability due to ether linkages in the main polymer backbones [44,45]. However, poly(phenylene)s particularly poly(2,5-bezophenone) s, exhibit exceptional mechanical properties as they contain entirely aromatic backbones with pendants groups [46]. Additionally, to improve the mechanical properties of the membranes, Ni (0) catalyzed C-C coupling polymerization process can be the most suitable way to achieve high molecular weighted polymers from poly(phenylene)s [47,48,49].

Therefore, this work is an attempt at the synthesis of sulfonyl imide-based proton exchange polymer membranes (SI-PBP) from 2,5-dichlorovenzophenone (DCBP) using the Ni (0) catalyzed C-C polymerization followed by the post imidization of the poly(benzophenone)s polymers. The DCBP monomer shows good reactivity in polymerization by electron-withdrawing groups. Additionally, the absence of the ether linkages in the SI-PBP membranes provides good dimensional and chemical stability. Sulfonyl imide groups promote the proton conductivity through super acidity compared to sulfonic acid groups. The pendant sulfonyl imide groups also greatly affect the membranes’ properties and attributes to make very distinct ionic channels for proton conduction throughout the polymer networks. Therefore, the SI-PBP membranes are anticipated to be a remarkable alternative to PFSA membranes in fuel cell applications.

## 2. Materials and Methods

### 2.1. Materials

Potassium permanganate, 2-5-Dichlorotoluene pyridine, thionyl chloride, carbon disulfide, benzene, aluminum chloride, chlorosulfuric acid, hydrochloric, nickel bromide, zinc powder, and triphenylphosphine were received from TCI, Sigma-Aldrich, and Alfa Aesar. Dimethylsulfoxide (DMSO), dimethylacetamide, dichloromethane, methanol, ethanol was used as received. The 2,5-Dichlorobenzophenone (DCBP) monomer, precursor’s fluorosulfonyl isocyanate (FSO_2_NCO), and sulfamoyl fluoride (FSO_2_NH_2_) were synthesized with high purity and yield (Schemes S1–S3 and Figures S1–S3, respectively).

### 2.2. Synthesis of Poly(Benzophenone) Polymers (PBP)

Inside in a glove box, a 3-necked catalytic flask was charged with (0.366 g, 1.19 mmol), zinc powder (4.54 g, 71.68 mmol), and triphenylphosphine (2.51 g, 9.56 mmol) and 2,5-dichlorobenzophenone (DCBP) monomer (3.0 g, 11.95 mmol) were charged into another flask. With a syringe, DMAc (3–5 mL) was added into the catalytic flask, fitted with a mechanical stirrer and nitrogen inlet/outlet. It was stirred gently at 80 °C until it turned a blood-red color. Thereafter, DCBP monomer was dissolved into DMAc solvent and added into the catalytic flask through a syringe. The resultant mixture was stirred 12 h at 100 °C to get a viscous dark mixture. The viscous mixture was diluted with 8–10 mL DMAc and cooled to normal temperature. Then, the solution was poured with stirring into the distilled water containing 30% HCl. When the white solids come out, the solids were collected through filtration and washed with distilled water and methanol, respectively. Finally, the white solid polymer (PBP) was dried 12 h in a vacuum oven at 60 °C.

### 2.3. Sulfonation of the PBP Polymer (SPBP)

At first, PBP polymers (2.0 g, 7.96 mmol) were dissolved in chloroform (10 mL) at 25 °C. The concentrated chlorosulfuric acid (3.18 mL, 47.8 mmol) was then added slowly in an ice condition and stirred for the required time at 80 °C. The black-colored solution was then poured into the ice water and stirred vigorously to obtain yellow solids. Finally, the yellow solid polymer was rinsed with distilled water and dried at 60 °C for 24 h.

### 2.4. Synthesis of Sulfonyl Imide Poly(Benzophenone)s Polymers (SI-PBP)

A 100 mL flask was charged with SPBP polymer (2.0 g, 5.66 mmol), 15 mL dichloromethane (15 mL), and thionyl chloride (20 mL). The mixture was reflux with 2 mL DMF at 75 °C for 24 h. Thereafter, all the solvents were evaporated to obtain a viscous solution. Diluting the resultant viscous solution with dichloromethane (15 mL), sulfamoyl fluoride (2.24 g, 22.65 mmol) was added dropwise in an ice condition. The mixture was stirred for 12 h at 25 °C. The resultant orange color solution was then poured into methanol to obtain the sulfonyl imide poly(benzophenone) polymers (SI-PBP). After collecting the polymer by filtration it was dried at 60 °C.

## 3. Results and Discussion

### 3.1. Preparation of Monomer (DCBP)

The 2,5-dichlorobenzophenone (DCBP) monomer was synthesized from 2,5-dichlorotoluene followed by Friedel-Craft acylation with benzene (Scheme S1). ^1^H-NMR (Nuclear Magnetic Resonance) was used to confirm the structure of the DCBP monomer (Figure S1). The chemical structure of the DCBP monomer was confirmed by ^1^H-NMR (Figure S1). All the phenyl protons appeared at 7.35–7.84 ppm. For the mesomeric effect of the chlorine atoms, the protons in the ortho and meta position appeared at upfield near 7.39–7.42 and 7.36 ppm, respectively. Additionally, the ortho, meta, and para protons of side phenyl rings shifted to the downfield because of the electron withdrawal effect of the carbonyl group and appeared at near 7.46–7.52, 7.60–7.66 and 7.78–7.85 ppm, respectively.

### 3.2. Preparation of Polymers (SI-PBP)

**Scheme 1:**
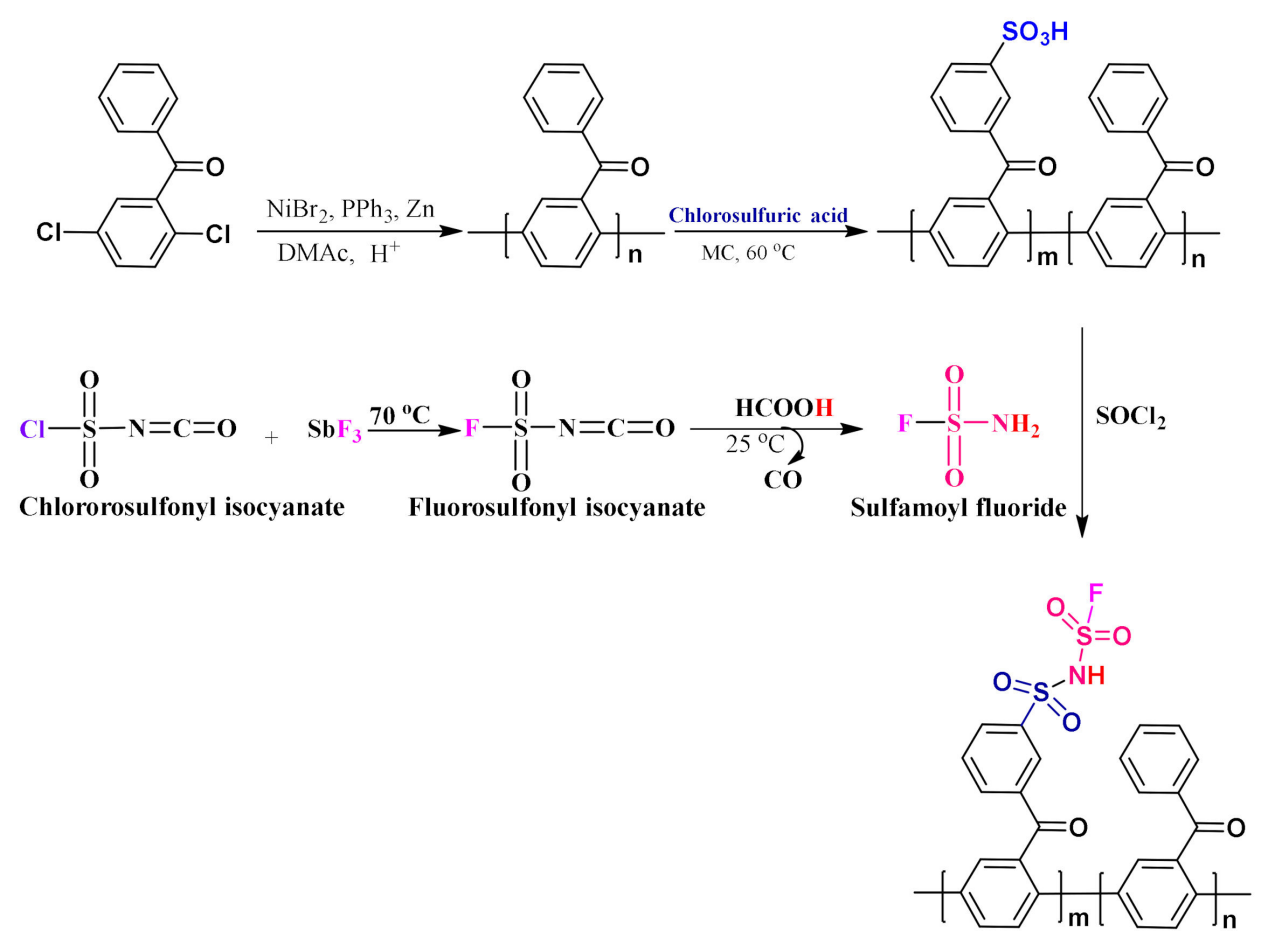
Synthesis route towards sulfonyl imide poly(benzophenone) polymers (SI-PBP).

**Figure 1:**
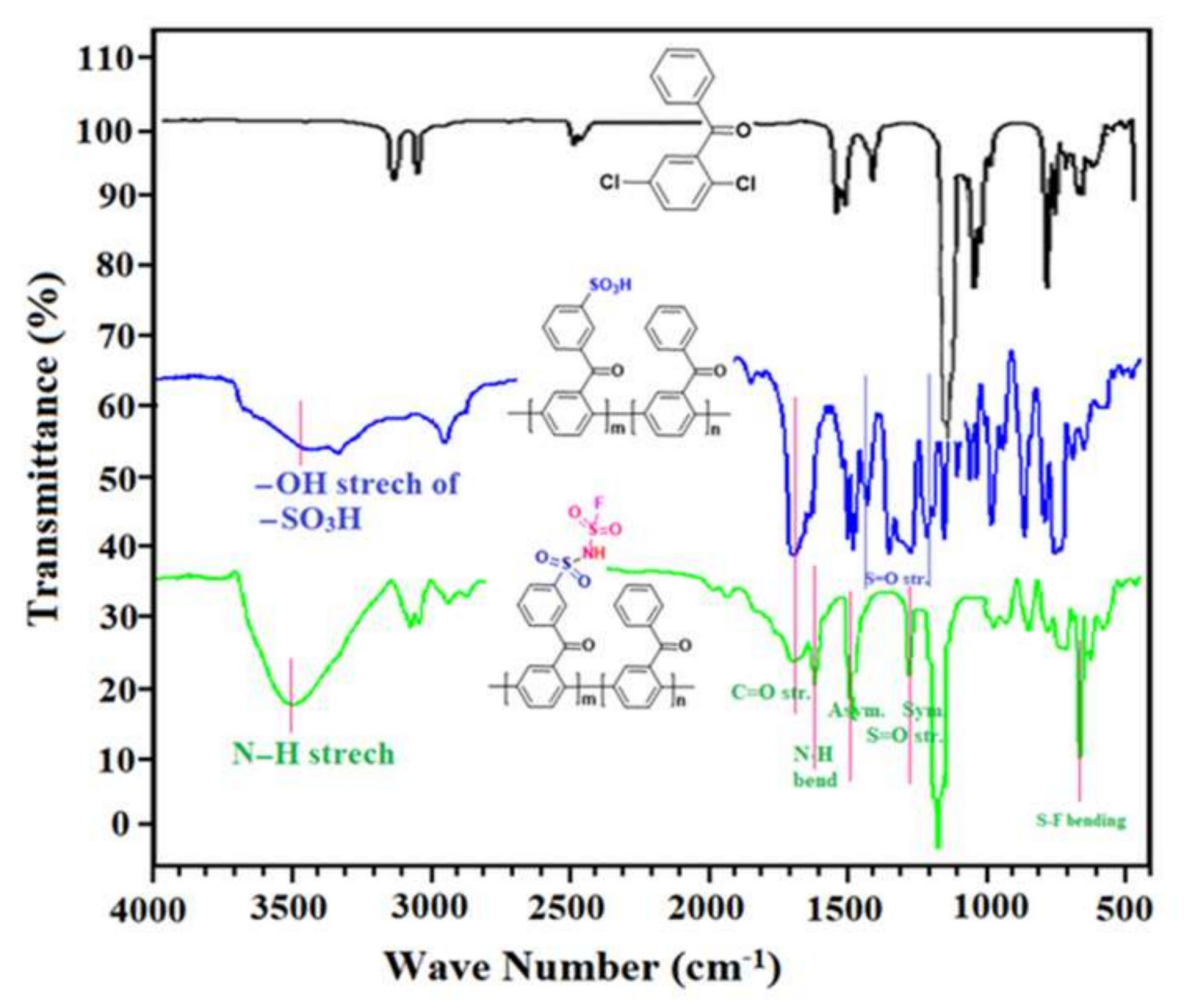
FT-IR spectra of PBP, S-PBP and SI-PBP.

**Figure 2:**
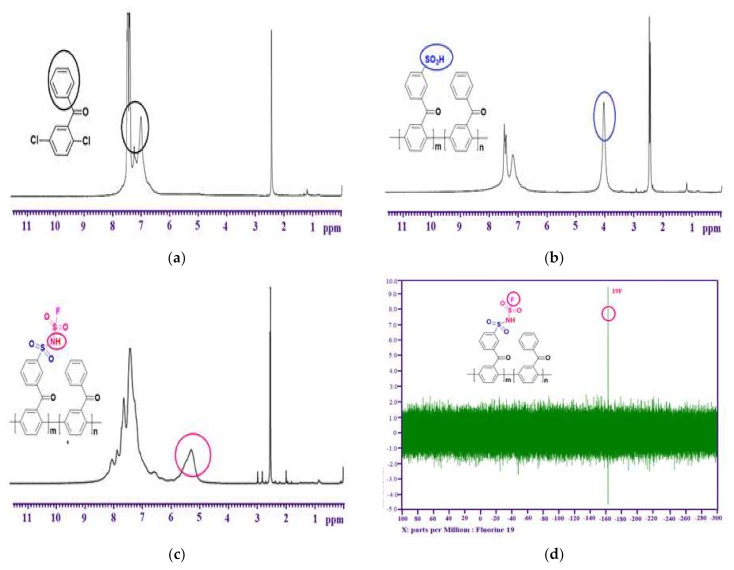
^1^H-NMR of (**a**) PBP (**b**) S-PBP and (**c**) SI-PBP polymers (**d**) ^19^F-NMR of the SI-PBP polymers.

The SI-PBP polymers were synthesized by C-C coupling polymerization of the DCBP monomer using Ni (0) catalyst (Scheme 1). The molar ratio of the catalyst plays an important role to get high molecular weighted polymers. Therefore, the molar ratio of the catalyst NiBr_2_: PPh_3_: Zn (1:10:40) was applied in the synthesis of the PBP polymers. Additionally, chlorosulfonation of PBP polymers (S-PBP) was acquired by controlling the amount and time of exposure to chlorosulfuric acid. Thereafter, sulfonimidization was carried out using SOCl_2_ and sulfamoyl fluoride (FSO_2_NH_2_), respectively. The SI-PBP polymers was characterized by FT-IR (Fourier Transform Infrared Spectrometer), ^1^H-NMR, and ^19^F-NMR (Figure 1 and Figure 2) respectively. In Figure 1, the broad stretching frequencies ranging from 2800–3600 cm^−1^ corresponds to the -OH vibrational frequencies of the S-PBP polymers. The stretching frequencies at 1420 and 1190 cm^−1^ correspond to S=O groups (assy. & sym.) whereas frequency at 1680 cm^−1^ for C=O bonds. However, the sharp characteristic peaks for N–H stretching found at 3500 cm^−1^ and N-H bending was overlapped with C=O stretching at 1680 cm^−1^ for the SI-PBP polymers. The peaks at 1480 and 1250 cm^−1^ respectively represent the asymmetric and symmetric stretching vibration of S=O groups for sulfonyl imide groups. Additionally, the frequencies at 640 cm^−1^ for S-F bonds confirms the sulfonyl imide incorporation to the sulfuric acid groups. Figure 2 shows the ^1^H-NMR and ^19^F-NMR for the PBP, SPBP and SI-PBP polymers. The peaks around 6.50–7.78 ppm correspond to the protons of the aromatic rings for PBP and SPBP polymers (Figure 2a,b). Again, the -SO_3_H peaks for SPBP polymers observed at the upfield at 4.0 ppm due to the conjugation with moisture and solvent DMSO (Figure 2b). However, the aromatic protons peaks for SI-PBP polymers shifted towards the downfield at 6.50–8.60 ppm, due to the delocalization capability of the sulfonyl imide anion (Figure 2c). The characteristic lumpy N-H peaks were observed at 4.90–5.70 ppm. The characteristic ^19^F peak at near −162 ppm also assured the attachment of the sulfonyl imide groups into the polymer backbones (Figure 2d).

### 3.3. IEC, Water Uptake and Dimensional Stability of Membranes

The details about the method of characterization and properties of the SI-PBP membranes have been discussed in the Characterizations and Measurement of Membranes’ Properties section in the supporting details.

**Figure 3:**
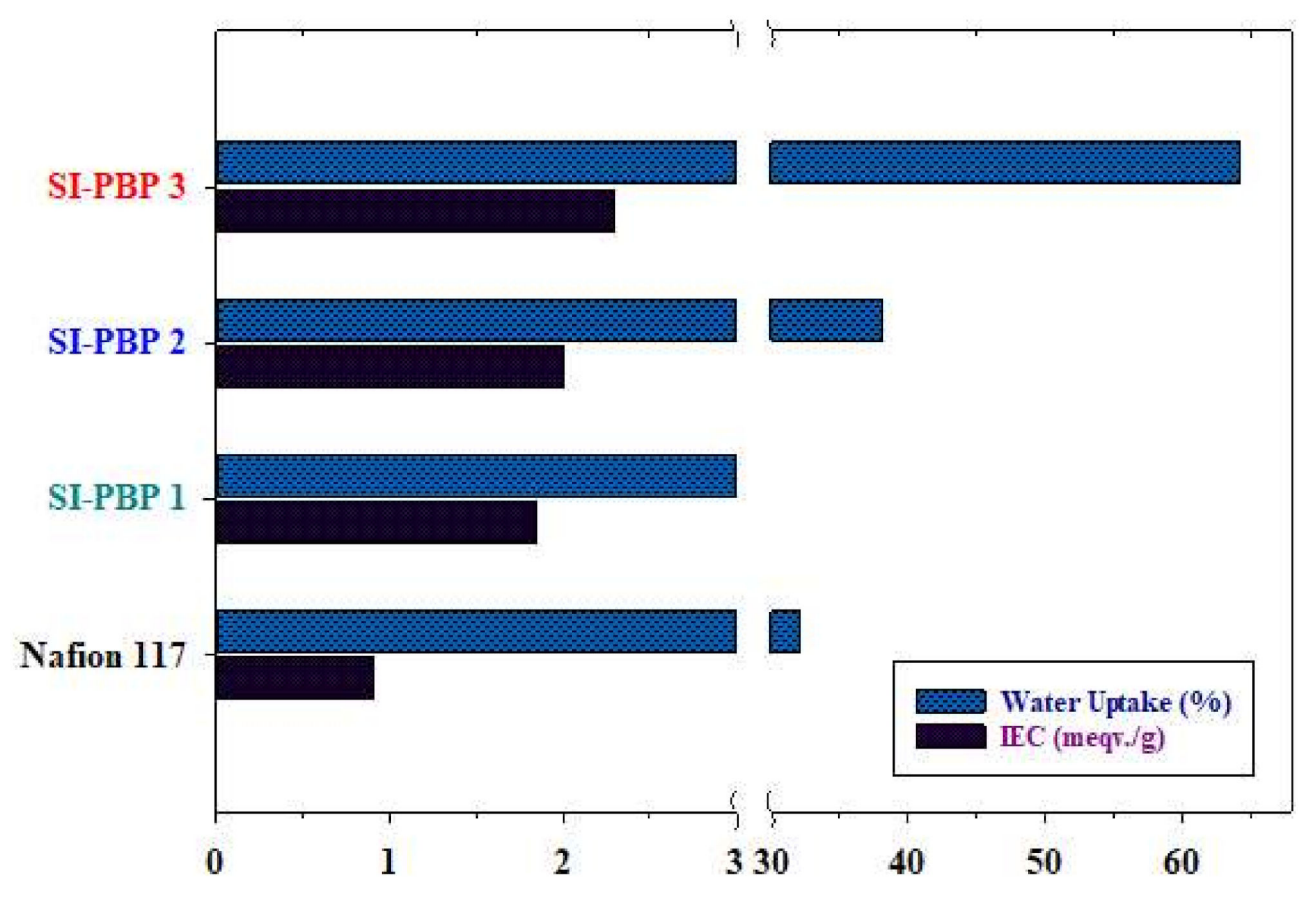
Ion exchange capacity (IEC) and Water uptake (WU) for the SI-PBP polymers membranes.

Figure 3 shows a gradual increase in the water uptake (22.72 to 64.29%) with the increment of IEC from 1.85–2.30 meq./g). The charge delocalization capability of the sulfonyl imide acid groups is accountable for the higher water content of the SI-PBP membranes.

The SI-PBP membranes show comparably lower-dimensional changes (Δt and Δl) than Nafion 211^®^. The SI-PBP membranes exhibited higher Δt (7.5%, 12.5%, and 34.0%) and Δl (4.81%, 8.0%, and 16.17%) values whereas Nafion 211^®^ showed (Δt = 31.98 and Δl = 14.2%) (Table 1). Noticeably, Δl values of SI-PBP membranes were significantly lower than the Δt values due to the C-C bonded aromatic rigid polymer backbones.

### 3.4. Proton Conductivity of the SI-PBP Membranes

**Figure 4:**
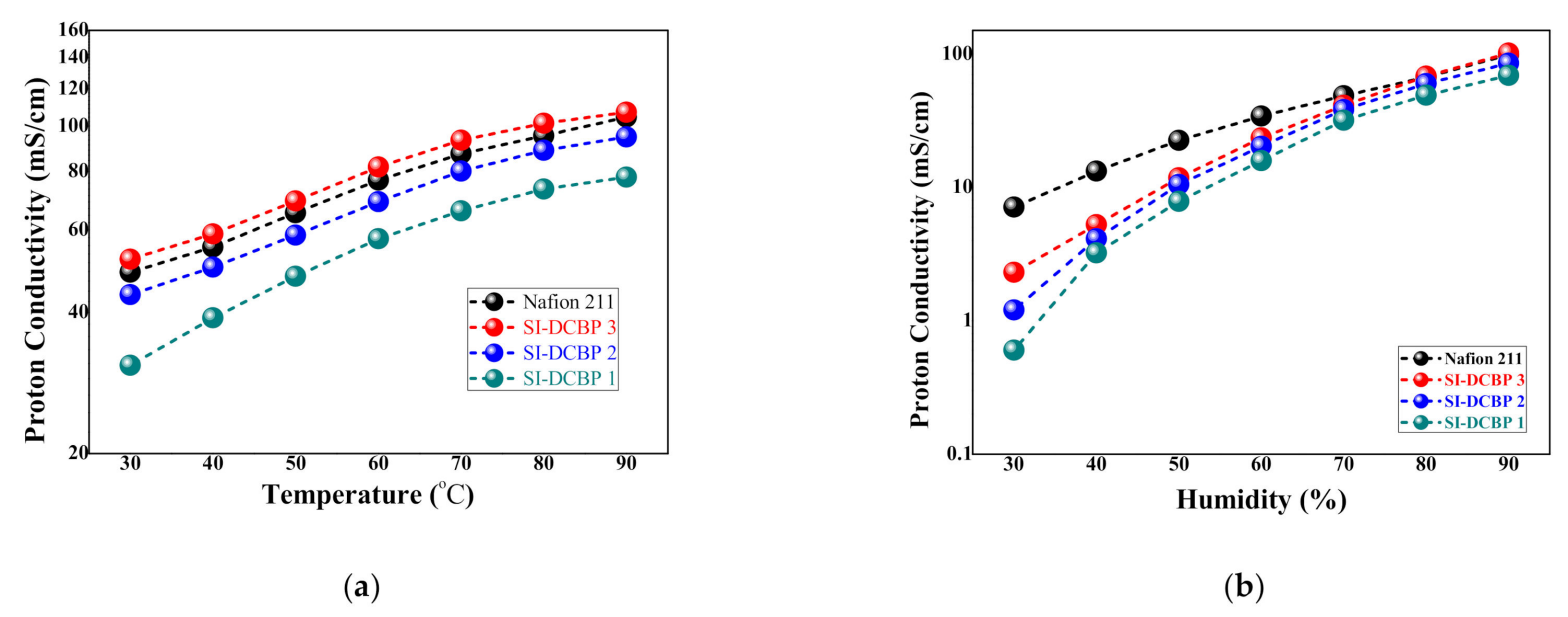
Proton conductivity for the SI-PBP polymer membranes in (**a**) different temperatures at 90% RH (**b**) different relative humidity at 90 °C.

Figure 4 represents the proton conductivity of the SI-PBP membranes at different temperatures (30–90 °C) and humidity (30–90% RH), respectively. Markedly, SI-PBP- 3 membrane showed significantly higher proton conductivity (107.07 mS/cm) than Nafion 211^®^ (104.5 mS/cm) under 90% RH at 90 °C (Figure 4a). Noticeably, SI-PBP 2 also exhibited comparable proton conductivity (94.80 mS/cm) to Nafion 211^®^ (104.5 mS/cm) in the same condition. However, the proton conductivity of the synthesized SI-PBP membranes was also increases with the humidity (Figure 4b). The higher acidity and location of the sulfonyl imide groups in the pendant benzophenone rings are mutually liable for the higher proton conduction of the SI-PBP membranes by forming well distinct ionic channels for proton conduction.

### 3.5. Thermo-Oxidative Stability of Membranes

**Figure 5:**
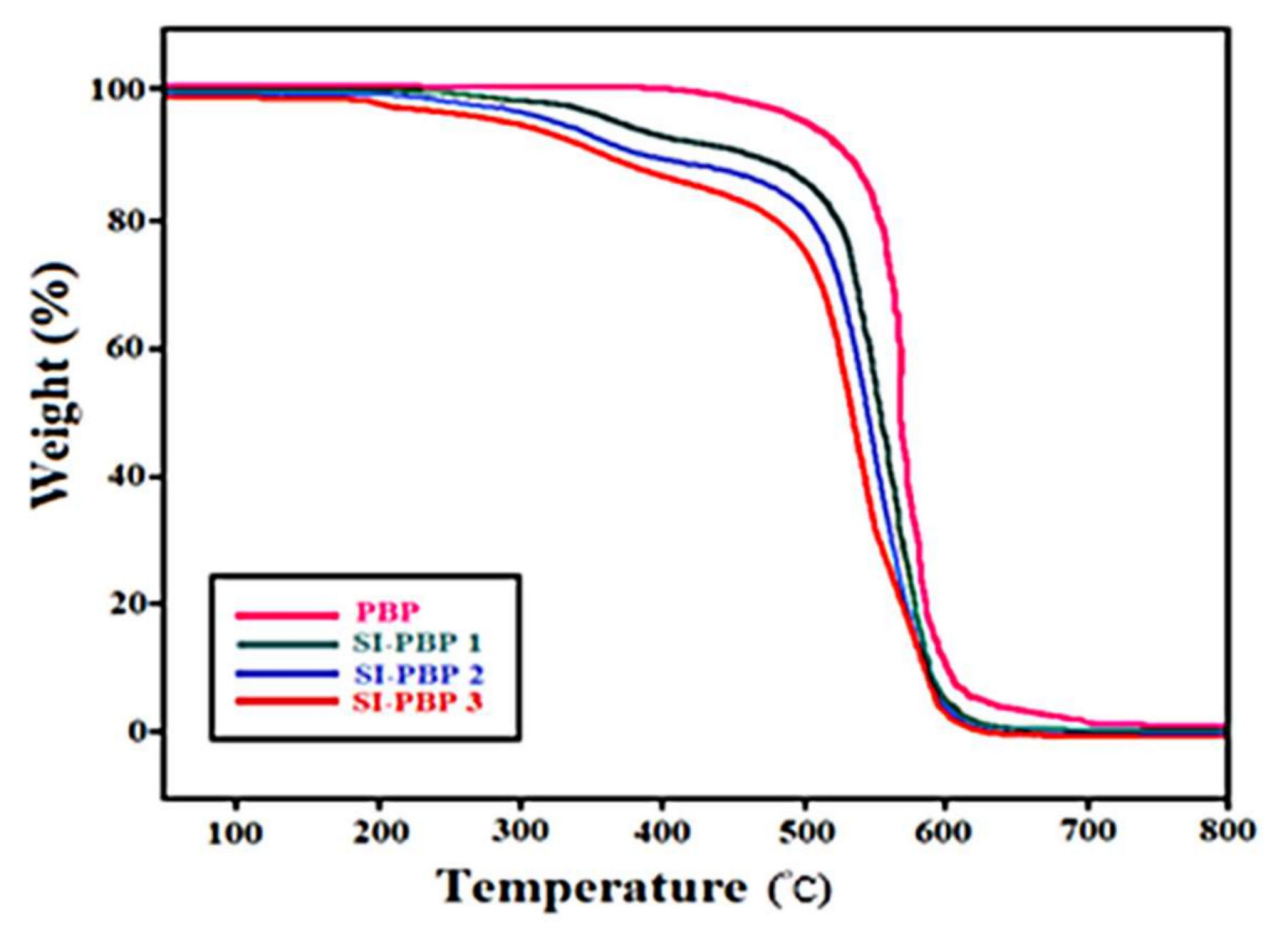
Thermo-oxidative stabilities of PBP and SI-PBP polymers.

Figure 5 depicts the thermal degradation curves for the SI-PBP membranes in an air atmosphere. The PBP polymer exhibited the thermal degradation in one step at around 380 °C whereas SI-PBP membranes showed three steps degradation. The thermal degradations at the ranges 200–330, 340–475 and 475–610 °C accounts for the conversion of the sulfonyl imide into sulfuric acid groups, removal of the -SO_3_H groups and polymer backbones degradation, respectively. Markedly, the most advantageous site of the SI-PBP polymers is the conduction of proton in two steps (i.e., with -SO_2_NHSO_2_- groups and -SO_3_H groups) before the breakdown of the main polymer backbones [43]. Additionally, the SI-PBP polymers are stable up to 550 °C. The C-C bonded aromatic backbones are mainly responsible for the extreme thermal stability of the SI-PBP membranes.

### 3.6. Chemical Stability of Membranes

**Figure 6:**
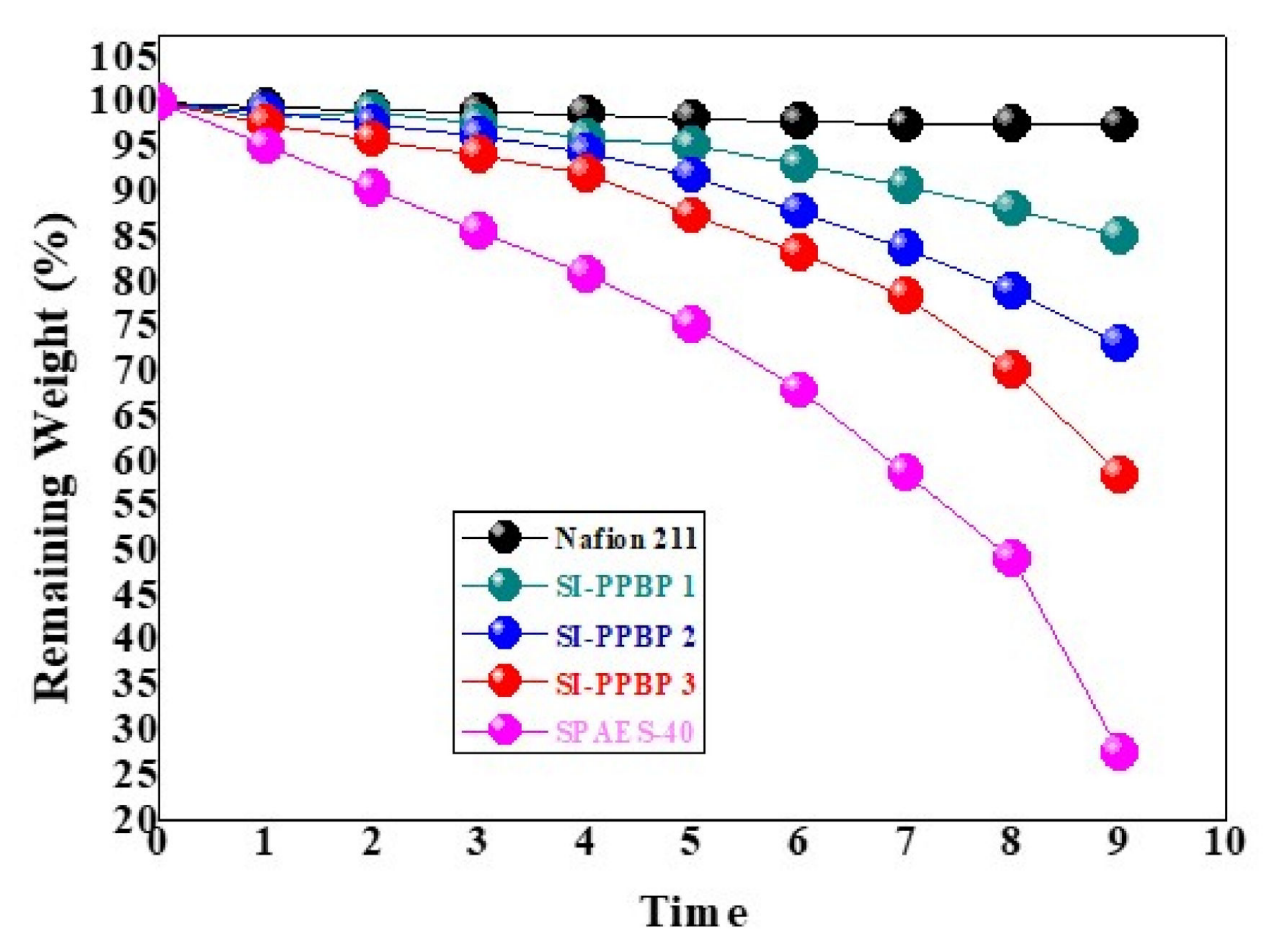
Fenton’s reagent (3 ppm Fe^2+^) test for the SI-PBP polymer membranes.

Figure 6 illustrates the chemical degradation of the SI-PBP membranes by Fenton’s reagent in terms of weight losses with times at 80 °C for 9 h. All the SI-PBP membranes showed excellent chemical stability compare to sulfonated poly (arylene ether sulfone), SPAES 40. The C-C polymer backbones which are less susceptibility towards free radical attacks and presence of the fluorine atoms in the sulfonyl imide groups are responsible for the higher chemical stability of the SI-PBP membranes.

### 3.7. Morphology of the Membranes

**Figure 7:**
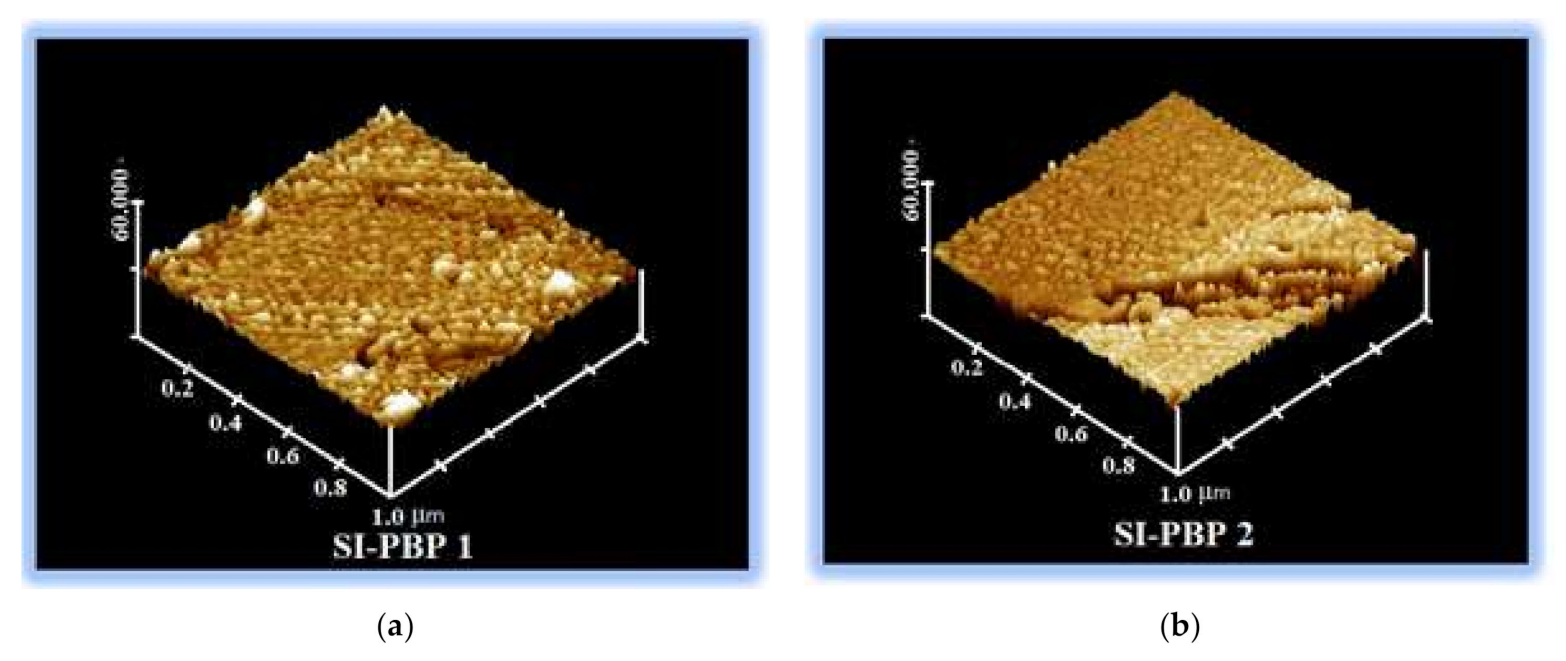

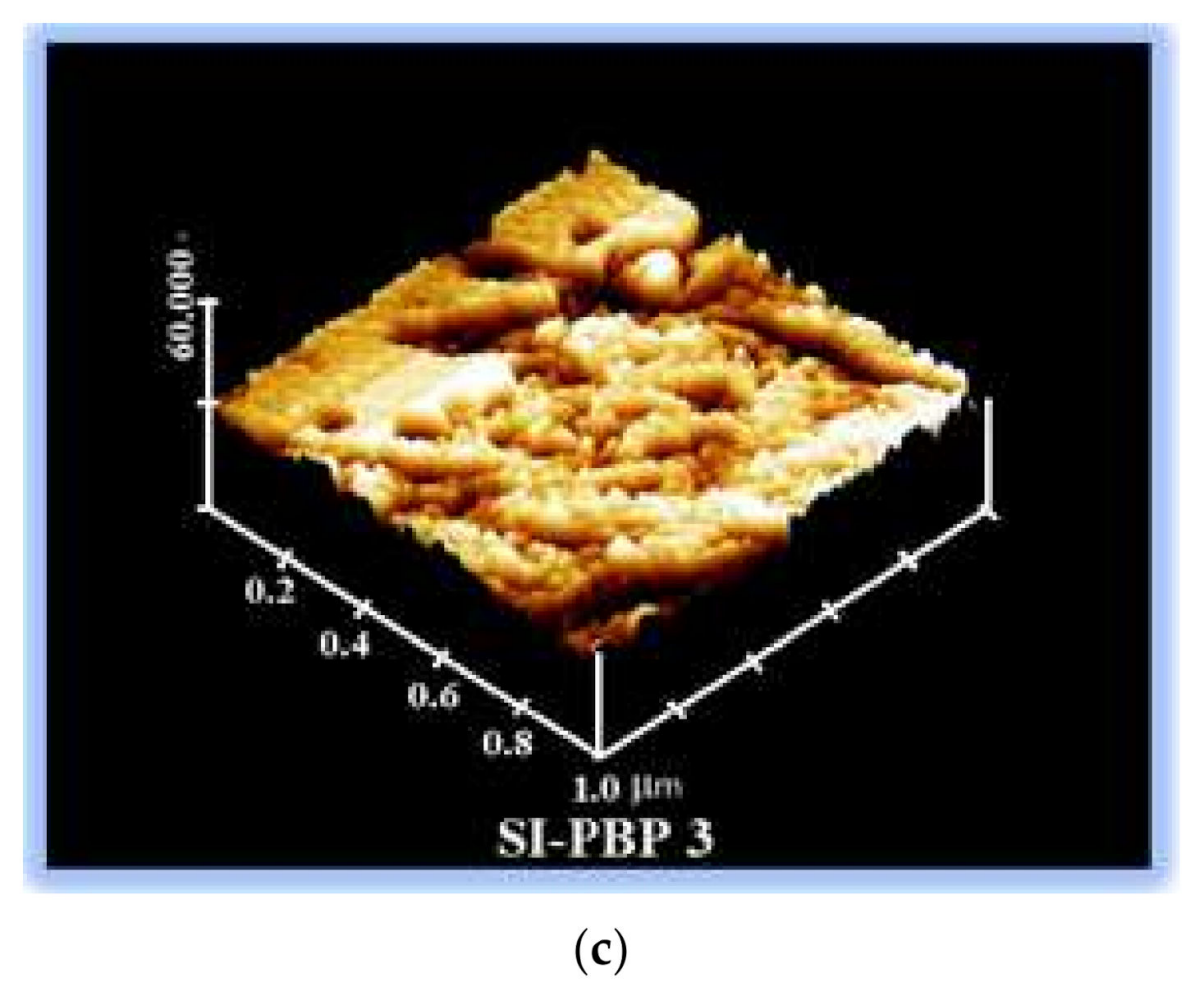
Atomic force microscopy images for (**a**) SI-PBP 1, (**b**) SI-PBP 2 and (**c**) SI-PBP 3 polymer membranes.

Figure 7 exemplifies the trapping mode surface views of the SI-PBP by atomic force microscopy (AFM). The brown segments represent the hydrophobic aromatic domains whereas the dark black channel-like portions indicate the hydrophilic sulfonyl imide groups together with moisture in the SI-PBP membranes. Typically, the polymer backbones and contents of the acid moiety greatly affect the morphology of the polymer membranes. Therefore, SI-PBP 3 shows a noticeable number of hydrophilic channels for containing a greater number of sulfonyl imide groups compare to the others. Conversely, SI-PBP 1 results interrupted ionic channels in the polymer membranes for having less sulfonyl imide groups.

### 3.8. Cell Performance of the SI-PBP Membranes

**Figure 8:**
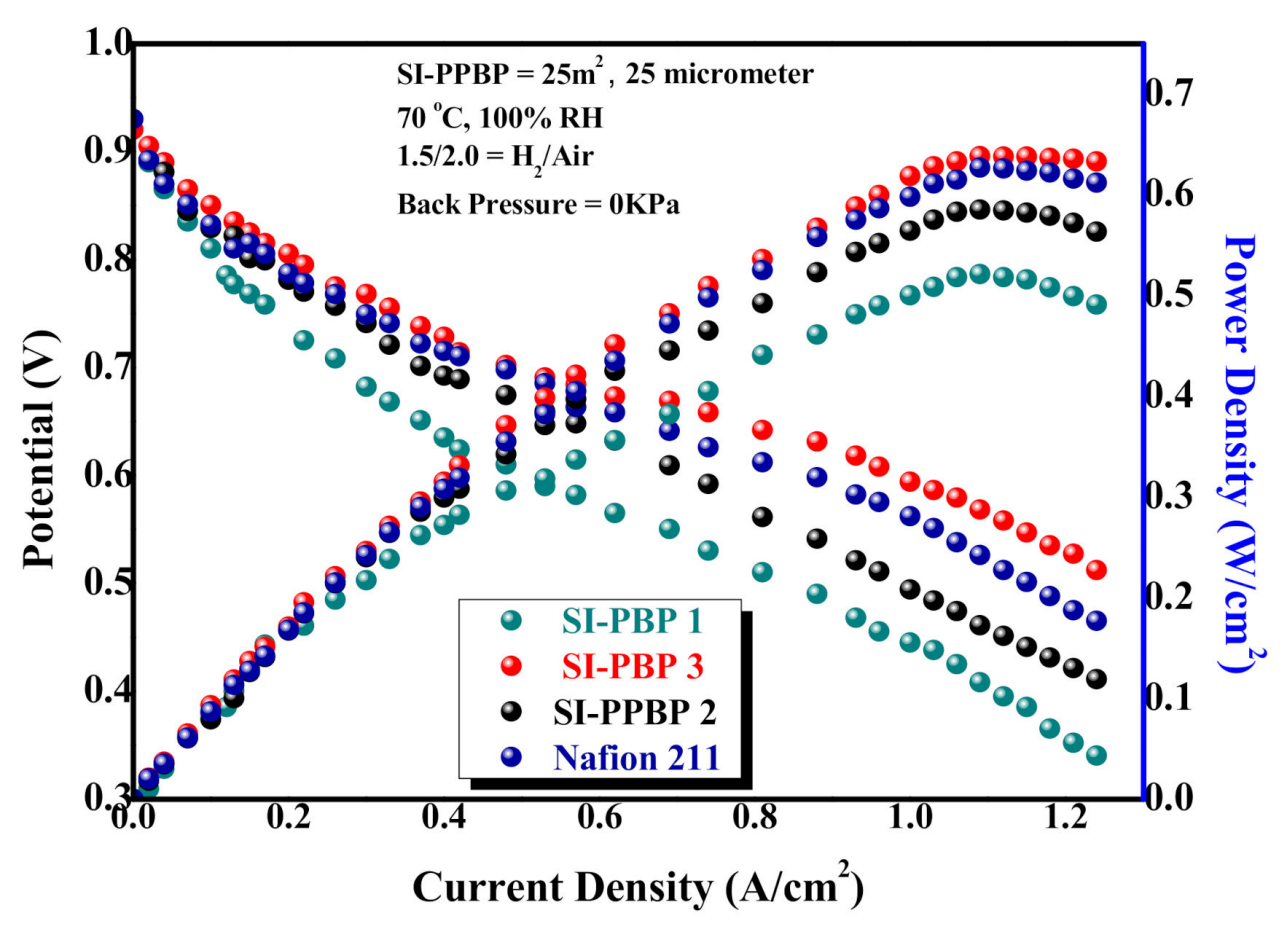
Cell performance for the SI-PBP polymer membranes.

Figure 8 illustrates the polarization curves for the single-cell performance of the SI-PBP membranes. The SI-PBP 3 membranes showed higher power density with a maximum power density (0.638 W/cm^2^) than Nafion 211^®^ (0.62 W/cm^2^). The SI-PBP 1 and SI-PBP 2 also displayed comparable cell performance as Nafion 211^®^ in the whole range of current density.

Dry membrane tensile stress strain properties are shown in Table 1. In dried membrane condition, the Young’s modulus of SPPBP-1,2 and 3 were 1005, 1014 and 1019 Mpa compared with 208 MPa of Nafion 211. These properties are momentous in PEMFC application, and make these membranes attractive for PEM materials.

## 4. Conclusions

The polymer membranes based on sulfonamide poly(benzophenone)s (SI-PBP) were synthesized by Ni (0) catalyzed C-C coupling polymerization of 2,5-dichloro-benzophenone. The synthesized SI-PBP membranes showed IECs from 1.85 to 2.30 meq./g and water uptake from 22.72 to 64.29%. The as-synthesized SI-PBP 3 membranes exhibited higher proton conductivity (107.07 mS/cm) with maximum power density (0.638 W/cm^2^) than Nafion 211^®^ (104.5 mS/cm and 0.62 W/cm^2^). The C-C coupled polymer backbones of the synthesized SI-PBP membranes provided outstanding thermal and chemical stability which are assured by the thermogravimetric analysis (TGA) and Fenton’s test. Additionally, during the cell operation, the presence of the fluorine atoms in the pendant side-chain protects the polymer backbones from free radical attacks. Moreover, the pendant sulfonyl imide groups in the benzophenone moiety provide well-defined ion-conducting channels for proton conduction throughout the polymer network. Hence, due to the excellent thermal, chemical stability with enhanced proton conductivity, the synthesized C-C coupled SI-PBP polymer membranes can be considered as an ultimate alternative to ether linkage based per fluorinated Nations.

## Figures and Tables

**Table 1:** Properties of membranes.

Polymers	Viscosity ^a^ η_int_ (dl/g)	IEC ^b^, (meq/g)	Water Content ^c^, (%)	Dimensional Changes ^c^		Hydration Number, Per Sulfonimide Group ^c^, λ	Conductivity Measurement ^d^, (mS/cm)		Young’s Modulus ^e^ (MPa)
				Δt (%)	Δl (%)		Membrans Thickness, L	σ	
SI-PBP 1	2.30	1.85	22.72	7.5	4.8	7.01	25 μm	72.75	1005
SI-PBP 2	2.12	2.00	38.09	12.5	8.0	10.58	25 μm	94.80	1014
SI-PBP 3	2.00	2.30	64.29	34.0	16.2	15.53	25 μm	107.07	1019
Nafion-211^®^	-	0.91	32.1	31.98	14.2	19.95	25 μm	104.5	208

^a^ Data obtained in DMSO at 25 °C. ^b^ Titrated with 0.01N NaOH. ^c^ Water Uptake and dimensional changes at 80 °C. ^d^ Proton Conductivity 90% RH (Relative Humidity) and 90 °C. ^e^ Dry condition.

## Data Availability

Data sharing is not applicable to this article.

## Supplementary Materials

The following are available online at https://www.mdpi.com/2077-0375/11/1/49/s1, Scheme S1: Synthesis route towards 2,5-dichlorobenzophenone monomer (DCBP), Scheme S2: Synthesis route for fluorosulfnyl isocyanate, Scheme S3: Synthesis route for Sulfamoyl fluoride, Figure S1: 1H NMR spectra of 2,5-dichlorobenzophenone monomer (DCBP), Figure S2: 19F-NMR spectra of fluorosulfonyl isocyanate (FSO2NCO), Figure S3: (a) 1H NMR, (b) ^19^F NMR of sulfamoyl fluoride (FSO_2_NH_2_).

## References

[1] Jang H., Hong T., Yoo J., Lee S., Ha J., Choi K., Lee C., Kim W. (2015). Synthesis and characterization of sulfonated polyphenylene containing benzophenone moiety via nickel catalyzed polymerization. Electrochim. Acta.

[2] Marx N., Boulon L., Gustin F., Hissel D., Agbossou K. (2014). A review of multi-stack and modular fuel cell systems: Interests, application areas and on-going research activities. Int. J. Hydrog. Energy.

[3] Zheng J., He Q., Gao N., Yuan T., Zhang S., Yang H. (2014). Novel proton exchange membranes based on cardo poly(arylene ether sulfone/nitrile)s with perfluoroalkyl sulfonic acid moieties for passive direct methanol fuel cells. J. Power Sources.

[4] Lin R., Weng Y., Lin X., Xiong F. (2014). Rapid cold start of proton exchange membrane fuel cells by the printed circuit board technology. Int. J. Hydrog. Energy.

[5] Ettihir K., Boulon L., Becherif M., Agbossou K., Ramadan H.S. (2014). Online identification of semi-empirical model parameters for PEMFCs. Int. J. Hydrog. Energy.

[6] Petrone R., Zheng Z., Hissel D., Péra M., Pianese C., Sorrentino M.C., Becherif M., Yousfi-Steiner N. (2013). A review on model-based diagnosis methodologies for PEMFCs. Int. J. Hydrog. Energy.

[7] Borup R., Meyers J., Pivovar B., Kim Y.S., Mukundan R., Garland N., Myers D., Wilson M., Garzon F., Wood D. (2007). Scientific Aspects of Polymer Electrolyte Fuel Cell Durability and Degradation. Chem. Rev..

[8] Ying Y., Kamarudin S.K., Masdar M.S. (2018). Silica-related membranes in fuel cell applications: An overview. Int. J. Hydrog. Energy.

[9] Farooqui U.R., Ahmad A.L., Hamid N.A. (2018). Graphene oxide: A promising membrane material for fuel cells. Renew. Sustain. Energy Rev..

[10] Shaari N., Kamarudin S.K. (2017). Graphene in electrocatalyst and proton conductiong membrane in fuel cell applications: An overview. Renew. Sustain. Energy Rev..

[11] Bakangura E., Wu L., Ge L., Yang Z., Xu T. (2016). Mixed matrix proton exchange membranes for fuel cells: State of the art and perspectives. Prog. Polym. Sci..

[12] Kim D.J., Jo M.J., Nam S.Y. (2015). A review of polymer–nanocomposite electrolyte membranes for fuel cell application. J. Ind. Eng. Chem..

[13] Liu B., Robertson G.P., Kim D.S., Guiver M.D., Hu W., Jiang Z. (2007). Aromatic Poly(ether ketone)s with Pendant Sulfonic Acid Phenyl Groups Prepared by a Mild Sulfonation Method for Proton Exchange Membranes. Macromolecules.

[14] Liu B., Robertson G.P., Kim D.-S., Sun X., Jiang Z., Guiver M.D. (2010). Enhanced thermo-oxidative stability of sulfophenylated poly(ether sulfone)s. Polymer.

[15] Alberti G., Costantino U., Casciola M., Ferroni S., Massinelli L., Staiti P. (2001). Preparation, characterization and proton conductivity of titanium phosphate sulfophenylphosphonate. Solid State Ionics.

[16] Sahu A.K., Pitchumani S., Sridhar P., Shukla A.K. (2009). Nafion and modified-Nafion membranes for polymer electrolyte fuel cells: An overview. Bull. Mater. Sci..

[17] Pourcelly G. (2011). Membranes for low and medium temperature fuel cells. State-of-the-art and new trends. Pet. Chem..

[18] Kang K., Kim D. (2019). Pendant dual-sulfonated poly(arylene ether ketone) multi-block copolymer membranes for enhanced proton conductivity at reduced water swelling. J. Membr. Sci..

[19] Liang J., Ge J., Wu K., Zhang Q., Wang J., Ye Z. (2020). Sulfonated polyaryletherketone with pendant benzimidazole groups for proton exchange membranes. J. Membr. Sci..

[20] Rikukawa M., Sanui K. (2000). Proton-conducting polymer electrolyte membranes based on hydrocarbon polymers. Prog. Polym. Sci..

[21] Yandrasits M., Lindell M., Komlev A., Fort E., Hamrock S., Peppin D.M., Kalstabakken K. (2018). Stability of Perfluoro Bis(Sulfonyl)Imide-Based Ionomers in Fuel Cell Membranes and Electrodes. ECS Trans..

[22] Sun S., Ling L., Xiong Y., Zhang Y., Li Z. (2020). Trifluoromethanesulfonimide-based hygroscopic semi-interpenetrating polymer network for enhanced proton conductivity of nafion-based proton exchange membranes at low humidity. J. Membr. Sci..

[23] Eikerling M., Paddison S.J., Zawodzinski T.A. (2002). Molecular orbital calculations of proton dissociation and hydration of various acidic moieties for fuel cell polymers. J. New Mat. Electrochem. Syst..

[24] Obadia M.M., Mudraboyina B.P., Serghei A., Phan T.N.T., Gigmes D., Drockenmuller E. (2014). Enhancing Properties of Anionic Poly(ionic liquid)s with 1,2,3-Triazolium Counter Cations. ACS Macro Lett..

[25] Morozova S.M., Shaplov A.S., Lozinskaya E.I., Mecerreyes D., Sardon H., Zulfiqar S., Suárez-García F., Vygodskii Y.S. (2017). Ionic Polyurethanes as a New Family of Poly(ionic liquid)s for Efficient CO_2_ Capture. Macromolecules.

[26] Porcarelli L., Shaplov A.S., Salsamendi M., Nair J.R., Vygodskii Y.S., Mecerreyes D., Gerbaldi C. (2016). Single-Ion Block Copoly(ionic liquid)s as Electrolytes for All-Solid State Lithium Batteries. ACS Appl. Mater. Interfaces.

[27] Jangu C., Savage A.M., Zhang Z., Schultz A.R., Madsen L.A., Beyer F.L., Long T.E. (2015). Sulfonimide-Containing Triblock Copolymers for Improved Conductivity and Mechanical Performance. Macromolecules.

[28] Ma Q., Xia Y., Feng W., Nie J., Hu Y.S., Li H., Huang X., Chen L., Armand M., Zhou Z. (2016). Impact of the functional group in the polyanion of single lithium-ion conducting polymer electrolytes on the stability of lithium metal electrodes. RSC Adv..

[29] Feng S., Shi D., Liu F., Zheng L., Nie J., Feng W., Huang X., Armand M., Zhou Z. (2013). Single lithium-ion conducting polymer electrolytes based on poly[(4-styrenesulfonyl)(trifluoromethanesulfonyl)imide] anions. Electrochim. Acta.

[30] Bouchet R., Maria S., Meziane R., Aboulaich A., Lienafa L., Bonnet J.P., Phan T.N.T., Bertin D., Gigmes D., Devaux D. (2013). Single-ion BAB triblock copolymers as highly efficient electrolytes for lithium-metal batteries. Nat. Mater..

[31] Meziane R., Bonnet J.P., Courty M., Djellab K., Armand M. (2011). Single-ion polymer electrolytes based on a delocalized polyanion for lithium batteries. Electrochim. Acta.

[32] Schaberg M.S., Abulu J.E., Haugen G.M., Emery M.A., O’Conner S.J., Xiong P.N., Hamrock S. (2019). New Multi Acid Side-Chain Ionomers for Proton Exchange Membrane Fuel Cells. ECS Trans..

[33] Savett S.C., Atkins J.R., Sides C.R., Harris J.L., Thomas B.H., Creager S.E., Pennington W.T., Desmarteau D.D. (2002). A Comparison of Bis[(perfluoroalkyl)sulfonyl]imide Ionomers and Perfluorosulfonic Acid Ionomers for Applications in PEM Fuel-Cell Technology. J. Electrochem. Soc..

[34] Kusoglu A., Vezzu K., Hegde G.A., Nawn G., Motz A.R., Sarode H.N., Haugen G.M., Yang Y., Seifert S., Yandrasits M.A. (2020). Transport and Morphology of a Proton Exchange Membrane Based on a Doubly Functionalized Perfluorosulfonic Imide Side Chain Perflourinated Polymer. Chem. Mater..

[35] Handbook of Fluoropolymer Science and Technology—Google Books. https://onlinelibrary.wiley.com/doi/abs/10.1002/9781118850220.ch8.

[36] Kutt A., Rodima T., Saame J., Raamat E., Maemets V., Kaljurand I., Koppel I.A., Garlyauskayte R.Y., Yagupolskii Y.L., Yagupolskii L.M. (2011). Equilibrium Acidities of Superacids. J. Org. Chem..

[37] Zhao W., Sun J. (2018). Triflimide (HNTf2) in Organic Synthesis. Chem. Rev..

[38] Appleby A.J., Velev O.A., LeHelloco J.-G., Parthasarthy A., Srinivasan S., DesMarteau D.D., Gillette M.S., Ghosh J.K. (1993). Polymeric Perfluoro Bis-Sulfonimides as Possible Fuel Cell Electrolytes. J. Electrochem. Soc..

[39] Razaq M., Razaq A., Yeager E., Desmarteau D.D., Singh S. (1989). Perfluorosulfonimide as an Additive in Phosphoric Acid Fuel Cell. J. Electrochem. Soc..

[40] Razaq M., Razaq A., Yeager E., Desmarteau D.D., Singh S. (1987). Oxygen electroreduction in perfluorinated sulphonyl imides. J. Appl. Electrochem..

[41] Porcarelli L., Manojkumar K., Sardon H., Llorente O., Shaplov A.S., Vijayakrishna K., Gerbaldi C., Mecerreyes D. (2017). Single Ion Conducting Polymer Electrolytes Based On Versatile Polyurethanes. Electrochim. Acta.

[42] Rehahn M., Schlüter A.-D., Wegner G. (1990). Soluble poly(para-phenylene)s, 3. Variation of the length and the density of the solubilizing side chains. Die Makromol. Chem. Macromol. Chem. Phys..

[43] Sutradhar S.C., Ahmed F., Ryu T., Yoon S., Lee S., Rahman M., Kim J., Lee Y., Kim W., Jin Y. (2019). A novel synthesis approach to partially fluorinated sulfonimide based poly (arylene ether sulfone) s for proton exchange membrane. Int. J. Hydrog. Energy.

[44] Rohan R., Pareek K., Chen Z., Cai W., Zhang Y., Xu G., Gao Z., Cheng H. (2015). A high performance polysiloxane-based single ion conducting polymeric electrolyte membrane for application in lithium ion batteries. J. Mater. Chem. A.

[45] Rehahn M., Schlüter A.-D., Wegner G., Feast W.J. (1989). Soluble poly(para-phenylene)s. 2. Improved synthesis of poly(para-2,5-di-n-hexylphenylene) via Pd-catalysed coupling of 4-bromo-2,5-di-n-hexylbenzeneboronic acid. Polymer.

[46] Wallow T.I., Novak B.M. (1991). In aqua synthesis of water-soluble poly(p-phenylene) derivatives. J. Am. Chem. Soc..

[47] Kuo A.-T., Shinoda W., Okazaki S. (2016). Molecular Dynamics Study of the Morphology of Hydrated Perfluorosulfonic Acid Polymer Membranes. J. Phys. Chem. C.

[48] Hagberg E.C., Olson D.A., Sheares V.V. (2004). Advances in Ni(0)-Catalyzed Coupling for the Synthesis of Polythiophenes and Polyphenylenes. Macromolecules.

[49] Liang X., Pan G., Xu L., Wang J. (2015). A modified decal method for preparing the membrane electrode assembly of proton exchange membrane fuel cells. Fuel.

