# Supplementary material for: Sulfonyl Imide Acid-Functionalized Membranes via Ni (0) Catalyzed Carbon-Carbon Coupling Polymerization for Fuel Cells
Source: Membranes (Basel). 2021 Jan 12;11(1):49. doi: 10.3390/membranes11010049 (PMC7828272; doi:10.3390/membranes11010049)
Supplement: Supplementary file 1 [file membranes-11-00049-s001.pdf]

membranes

MDPI

Supplementary Materials

Supplementary Materials: Sulfonyl Imide Acid-Functionalized Membranes via Ni (0) Catalyzed Carbon-Carbon Coupling Polymerization for Fuel Cells

Sabuj Chandra Sutradhar [1,†], Sujin Yoon [1,†], Taewook Ryu [1], Lei Jin [1], Wei Zhang [1], Hohyoun Jang [2] and Whangi Kim [1,*]

[1] Department of Applied Chemistry, Konkuk University, Chungju, 27478, Republic of Korea; sabujchandra@gmail.com (S.C.S.); ysj920126@naver.com (S.Y.); gundam0924@naver.com (T.R.); jinlei8761@naver.com (L.J.);
arno_zw@hotmail.com (W.Z.)
[2] Department of Liberal Art, Konkuk University, Chungju, 27478, Republic of Korea; 200417450@kku.ac.kr
* Correspondence: wgkim@kku.ac.kr; Tel.: +82-43-840-3579 (ext. 410)
† These authors contributed equally to this work.

1. Scheme

Scheme S1. Synthesis route towards 2,5-dichlorobenzophenone monomer (DCBP).

Scheme S2. Synthesis route for fluorosulfnyl isocyanate

Scheme S3. Synthesis route for Sulfamoyl fluoride

2. Figures

Figure S1. ^{1}H NMR spectra of 2,5-dichlorobenzophenone monomer (DCBP).

Figure S2. ^{19}F-NMR spectra of fluorosulfonyl isocyanate (FSO_2NCO).

Figure S3. (a) 1H NMR, (b) 19F NMR of sulfamoyl fluoride (FSO2NH2).

3. Characterizations and Measurement of Membranes Properties

1. Scheme

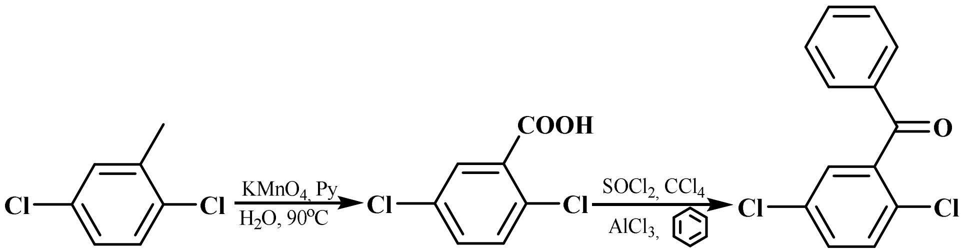

Scheme S1. Synthesis route towards 2,5-dichlorobenzophenone monomer (DCBP).

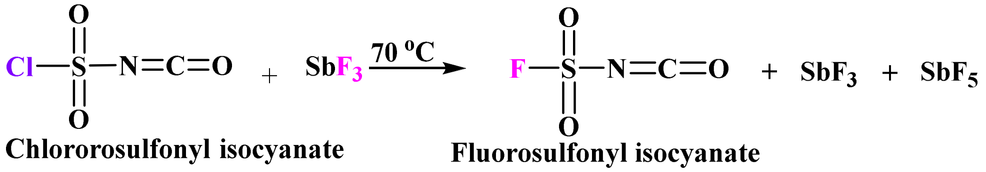

Scheme S2. Synthesis route for fluorosulfnyl isocyanate.

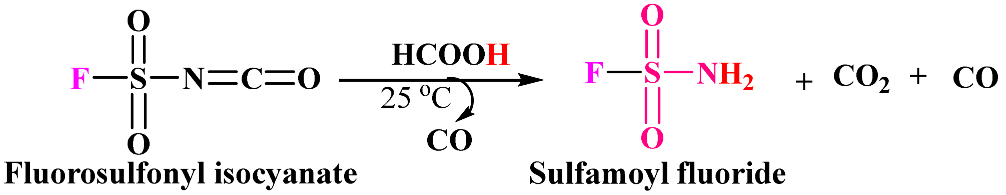

Scheme S3. Synthesis route for Sulfamoyl fluoride.

2. Figures

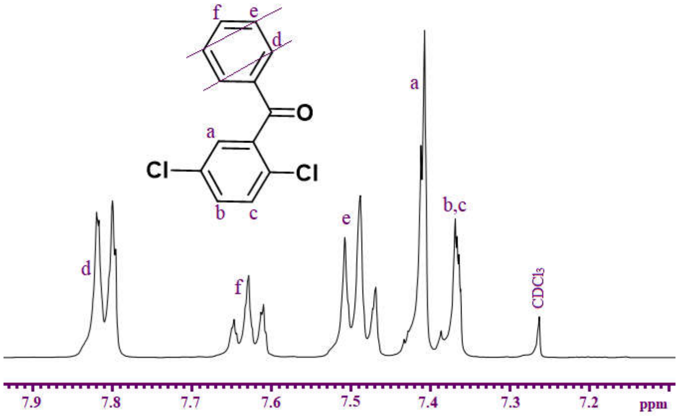

Figure S1. ^{1}H NMR spectra of 2,5-dichlorobenzophenone monomer (DCBP).

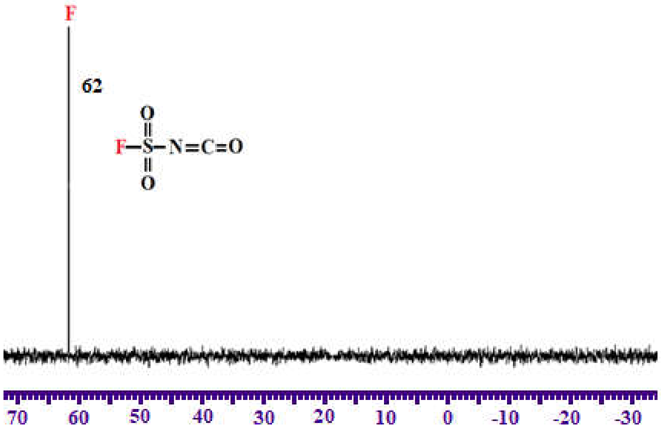

Figure S2. ^{19}F-NMR spectra of fluorosulfonyl isocyanate (FSO_2NCO).

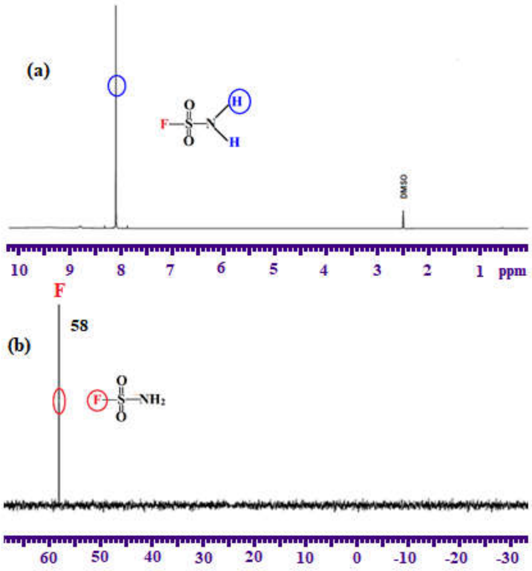

Figure S3. (a) ^{1}H NMR, (b) ^{19}F NMR of sulfamoyl fluoride (FSO_2NH_2).

3. Characterizations and Measurement of Membranes Properties

The membranes of 25 μm thickness were made by dissolving 3% w/v SI-PBP polymers in the DMAc solvent followed by exposing overnight under IR lamp on a glass plate. Typically, RheoSense, hts-VROC™ viscometer was used to measure the viscosity of the SI-PBP polymers. The structural properties of the synthesized DCBP monomer and SI-PBP polymers were studied using JEOL (400 YH) for ^{1}H and ^{19}F-NMR and FTIR spectra with Nicolet iS5 FTIR Spectrometry (Serial no. ASB1100426).

Usually, constant weighted polymer membranes were immersed into distilled water for 24 h at 80 ℃ and measured the water uptake as follows:

Water uptake,

$$WU\ (\%) = \{(W_{wet} - W_{dry}) / W_{dry}\} \times 100\% \quad (1)$$

Copyright: © 2021 by the authors. Licensee MDPI, Basel, Switzerland. This article is an open access article distributed under the terms and conditions of the Creative Commons Attribution (CC BY) license (http://creativecommons.org/licenses/by/4.0/).

where W_{dry} and W_{wet} imply the weight membranes at dried and wet conditions, respectively.

However, the SI-PBP membranes were stirred into a 1N NaCl solution for 24h at 80 ℃ to exchange the H^+ ions with Na^+. Subsequently, the exchanged H^+ was evaluted by titration with 0.01N NaOH solution to measure the ion exchange capacity (IEC) as follows:

$$IEC\ (meq./g) = (V_{NaOH} \times M_{NaOH}) / Wt_{dry}\ membrane \quad (2)$$

where, V_{NaOH}, M_{NaOH}, and Wt_{dry} correspond to the volume, molarity of the NaOH, and weight of the membrane respectively.

Consequently, others membrane properties i.e. hydration number (λ) and dimensional changes were also evaluated as follows:

$$\lambda = (10 \times WU\ \%) / (IEC \times 18) \quad (3)$$

$$\Delta l\ (\%) = \{(l_{wet} - l_{dry}) / l_{dry}\} \times 100 \quad (4)$$

$$\Delta t\ (\%) = \{(t_{wet} - t_{dry}) / t_{dry}\} \times 100 \quad (5)$$

where, λ, l and t represent the hydration number, length, and thickness of the membranes, respectively.

The through-plane conductivity of the SI-PBP membranes was conducted using MTS 740 membrane test system (Scribner Associates Inc., Southern Pines, NC, USA) with a Newton 4th Ltd. (N4L) impedance analysis interface (PSM 1735). Constant alternating current was applied through both electrodes having the membranes in the middle while maintaining the specific humidity (30~90%) and temperature (30~90℃) during the operation . Usually, the conductivity of the membranes was evaluated following the eq.:

$$\sigma = [L / (R_{mem} \times A)] \quad (6)$$

where L, A, and R_{mem} denote the thickness, electrode area, and corresponding resistance of the membranes, respectively.

The thermal property of the SI-PBP polymer membranes was investigated with TGA-N 1000 analyzer (Scinco, Chicago, IL, USA) at 30–800 ℃ with a scan rate of 20 ℃/min under air conditions.

Additionally, the chemical stability of the membranes was evaluated by heating the membranes into Fenton's reagent (3 ppm Fe^{2+}, 3% H_2O_2) at 80 ℃ and recorded the chemical degradation by measuring the weight of the membranes with 1h time interval up to 9h.

The hydrophilic and hydrophobic phase separation of the membrane was assessed by the trapping mode atomic force microscopy (AFM) with Nanoscope (R) IIIA, using microfabricated cantilevers with an amplitude setpoint 0.7785 V.

Membrane electrode assemblies (MEAs) with an active areaof 25 cm^2 were prepared using a decal method based on a catalyst coated membrane (CCM). A 20 wt% wet-proofed Toray carbon paper (TGPH-060, Toray Inc.) of 190 mm in thickness was employed as a gas diffusion layer (GDL) for the anode and cathode sides. Carbon-supported Pt (Hispec 13100, Johnson Matthey Inc.) was used as catalyst for both anode and cathode and the loading of the catalyst layer in this work was 0.29 mg Pt/cm^2. Immediately afterwards, the catalyst layer was transferred on the membrane at 120 ℃ and 10 MPa for 5 min by decal method to make the CCM. The GDL was placed on the anode and cathode side of the CCM to form the MEAs. After assembling the single cell, the MEAs were fully hydrated by feeding a fully humidified N_2 into the single cell for 2 h. During the operation, fully humidified H_2 and air at 70 ℃ were fed into the anode and cathode, respectively. The stoichiometry of hydrogen and to air was maintained to be 1.5/2.0 and the relative humidity 100/100%. After the activation procedure, polarization curves were measured with a commercial test station (Scitech, Korea Inc) at the temperature of 70 ℃ and ambient pressure. Polarization measurements were started at the OCV and the cell was operated in the galvanostatic mode with a scan rate of 36 mA/s for each step. The tensile stress-strain properties of the membranes were tested using a Com-Ten Industries 95T series load frame equipped with a 200 lbf load cell and computerized data acquisition software. Samples of 9 mm width were deformed at a crosshead speed of 5 mm/min with gauge length of 30 mm.
